# Aging is associated with increased FGF21 levels but unaltered FGF21 responsiveness in adipose tissue

**DOI:** 10.1111/acel.12822

**Published:** 2018-07-24

**Authors:** Joan Villarroya, José M. Gallego‐Escuredo, Alejando Delgado‐Anglés, Montserrat Cairó, Ricardo Moure, Ma Gracia Mateo, Joan C. Domingo, Pere Domingo, Marta Giralt, Francesc Villarroya

**Affiliations:** ^1^ Infectious Diseases Unit, Hospital de la Santa Creu i Sant Pau Barcelona Catalonia Spain; ^2^ Departament de Bioquímica i Biomedicina Molecular and Institut de Biomedicina (IBUB) Universitat de Barcelona Barcelona Spain; ^3^ Institut de Recerca Biomèdica (IRB) de Lleida Lleida Spain; ^4^ CIBER Fisiopatología de la Obesidad y Nutrición Barcelona Spain; ^5^ Department of Infectious Diseases Hospital Universitari Arnau de Vilanova Lleida Spain; ^6^ Department of Infectious Diseases Hospital Universitari de Santa María Lleida Spain; ^7^ Universitat de Lleida Lleida Spain

## Abstract

Fibroblast growth factor 21 (FGF21) has been proposed to be an antiaging hormone on the basis of experimental studies in rodent models. However, circulating FGF21 levels are increased with aging in rodents and humans. Moreover, despite the metabolic health‐promoting effects of FGF21, the levels of this hormone are increased under conditions such as obesity and diabetes, an apparent incongruity that has been attributed to altered tissue responsiveness to FGF21. Here, we investigated serum FGF21 levels and expression of genes encoding components of the FGF21‐response molecular machinery in adipose tissue from healthy elderly individuals (≥70 years old) and young controls. Serum FGF21 levels were increased in elderly individuals and were positively correlated with insulinemia and HOMA‐IR, indices of mildly deteriorated glucose homeostasis. Levels of β‐Klotho, the coreceptor required for cellular responsiveness to FGF21, were increased in subcutaneous adipose tissue from elderly individuals relative to those from young controls, whereas FGF receptor‐1 levels were unaltered. Moreover, total ERK1/2 protein levels were decreased in elderly individuals in association with an increase in the ERK1/2 phosphorylation ratio relative to young controls. Adipose explants from aged and young mice respond similarly to FGF21 “ex vivo”. Thus, in contrast to what is observed in obesity and diabetes, high levels of FGF21 in healthy aging are not associated with repressed FGF21‐responsiveness machinery in adipose tissue. The lack of evidence for impaired FGF21 responsiveness in adipose tissue establishes a distinction between alterations in the FGF21 endocrine system in aging and chronic metabolic pathologies.

Fibroblast growth factor 21 (FGF21) is a member of the endocrine FGFs subfamily. Pharmacological treatment with FGF21 ameliorates age‐related metabolic disorders such as insulin resistance, dyslipidemia, and obesity in rodents (Coskun et al., 2008; Kharitonenkov et al., 2005), and pilot studies in humans indicate that treatment with an FGF21‐analog has beneficial effects on hyperlipidemia and body weight (Gaich et al., 2013). Sustained increases in FGF21 levels attained by transgenic overexpression of FGF21 extend the lifespan of mice (Zhang et al., 2012), suggesting that FGF21 is a pro‐longevity hormone. Circulating FGF21 levels in humans increase with age from 5 to 80 years in healthy individuals independently of body composition (Hanks et al., 2015). In contradiction, low levels of FGF21 are related to healthy aging in centenarians (Sanchis‐Gomar et al., 2015). In addition, endurance exercise in elderly individuals reduces FGF21 levels (Taniguchi, Tanisawa, Sun, Kubo, & Higuchi, 2016). Thus, it has been suggested that the increases in FGF21 that parallel aging are related to the appearance of an age‐related FGF21‐resistant state, as has been proposed in metabolic diseases (Salminen, Kaarniranta, & Kauppinen, 2017). In obese and diabetic patients, FGF21 levels are abnormally elevated and an FGF21‐resistant state has been claimed to accompany these pathologies (Fisher et al., 2010). FGF21 is produced primarily in the liver and targets several tissues, mainly adipose tissue but also the brain and heart. The effects of FGF21 are mediated through interactions with FGF receptors on cell surface and require the coreceptor β‐Klotho (Ogawa et al., 2007). In fact, expression of β‐Klotho determines the specific responsiveness of cells to FGF21 and FGF19, another endocrine FGF that originates in the intestine. β‐Klotho expression and the ratio of phosphorylated ERK (extracellular signal‐regulated kinase) to total ERK, a marker of the extent of intracellular signaling driven by FGF21, are reduced in adipose tissues of obese patients with type II diabetes (Gallego‐Escuredo et al., 2015).

In this study, we analyzed FGF21 levels and alterations in the expression of genes encoding components of the FGF21‐responsive molecular machinery in adipose tissue from aged individuals so as to ascertain whether altered FGF21 responsiveness that develops with aging jeopardizes human health and/or accelerates metabolic disturbances associated with aging.

To this end, we studied a cohort of 28 healthy elderly individuals (≥70 years) with no overt signs of metabolic or other pathologies and compared them with a cohort of 35 young healthy controls (≤40 years). Serum FGF21 levels were significantly increased in elderly individuals compared with young healthy controls (Figure 1a). This is in line with previous reports describing an increase in FGF21 levels with aging (Hanks et al., 2015). The levels of FGF19, an endocrine FGF whose effects are also mediated by β‐Klotho, were unaltered with aging. Thus, the inverse relationship observed between FGF21 and FGF19 in obesity and diabetes—upregulation of FGF21 and downregulation of FGF19—(Gallego‐Escuredo et al., 2015) did not occur in healthy elderly individuals, despite the significant increase in FGF21 (Figure 1a). Despite the absence of overt diabetes, our population of aged individuals showed an increase in glycemia and insulinemia relative to young individuals (Table 1), an observation previously reported and considered an indication of impaired functionality of general metabolic homeostasis with age (Barzilai, Huffman, Muzumdar, & Bartke, 2012). In fact, FGF21 levels were positively correlated with glucose levels (*r* = 0.197, *p* = 0.048), insulin levels (*r* = 0.224, *p* = 0.038), and insulin resistance, measured as HOMA‐IR, (*r* = 0.427, *p* = 0.005) in the studied cohort.

**Figure 1:**
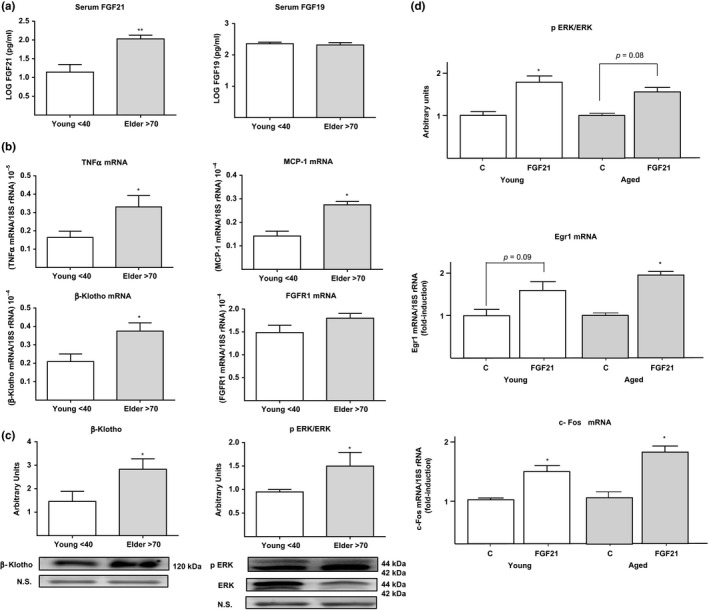
(a) Serum levels of FGF21 (left) and FGF19 (right) in young healthy controls (≤40) and elderly individuals (≥70). Serum levels of FGF21 and FGF19 are log‐transformed. (b) TNFα, MCP‐1, β‐Klotho, and FGFR1 mRNA expression in subcutaneous adipose tissue from young healthy controls and elderly individuals. Values are expressed relative to 18S rRNA (means ± *SEM*). (c) Levels of β‐Klotho protein (left) and ERK1/2 phosphorylation (right) in subcutaneous adipose tissue from young healthy controls and elderly individuals. Signal intensity was determined by densitometric quantitation of protein bands in immunoblot images (six individual samples per group). Phospho‐ERK1/2 levels were expressed relative to total ERK1/2. Membranes were stained with Coomassie Blue to normalize the amount of protein loaded. (d) Levels of ERK1/2 phosphorylation, Egr1 mRNA, and c‐Fos mRNA in mouse adipose tissue explants from young (5‐month‐old) and aged (16‐month‐old) mice treated with 30 nM FGF21 (four mice, triplicate explant analysis per mouse) and nontreated controls (c). All data are presented as means ± *SEMs*. *p < 0.05 and **p < 0.01 for comparisons between young healthy controls and elderly individuals, and FGF21‐treated vs. nontreated adipose explants

**Table 1:** Demographic, body composition, metabolic, and inflammation markers

	Control C (*n* = 35)	Elderly (>70) (*n* = 28)	*p* value
Sex (*n* of men (%))	23 (66.6)	14 (50)	0.193
Age (years)	38.1 ± 0.6	80.8 ± 1.1	**<0.001**
Weight (kg)	74.8 ± 5.0	70.8 ± 2.5	0.859
BMI	24.4 ± 0.6	27.4 ± 0.6	0.161
Waist‐to‐hip ratio	0.86 ± 0.03	0.92 ± 0.01	0.083
Total body fat (%)	26.3 ± 2.1	37.9 ± 1.4	**0.002**
Glucose (mmol/L)	4.73 ± 0.15	5.93 ± 0.23	**0.048**
Insulin (pmol/L)	38.7 ± 8.9	123.8 ± 18.6	**0.023**
HOMA‐IR	0.45 ± 0.11	1.81 ± 0.28	**0.011**
Bilirubin (mmol/L)	8.00 ± 1.32	12.69 ± 0.89	0.461
AST (U/L)	28.4 ± 2.6	22.1 ± 1.3	0.579
ALT (U/L)	40.9 ± 8.9	19.2 ± 1.5	**0.007**
Triglycerides (mmol/L)	1.07 ± 0.21	1.21 ± 0.11	0.856
Total cholesterol (mmol/L)	4.68 ± 0.31	5.08 ± 0.16	0.813
HDL cholesterol (mmol/L)	1.36 ± 0.07	1.33 ± 0.06	0.951
LDL cholesterol (mmol/L)	2.91 ± 0.32	3.17 ± 0.14	0.852
TNFα (pg/ml)	2.91 ± 0.28	4.96 ± 0.61	**0.003**
MCP−1 (pg/ml)	119.9 ± 7.0	252.1 ± 30.3	**<0.001**

Note

Parameters are expressed as mean ± *SEM* unless specified. *P* values were calculated using Student's *t* test for parametric data. Bold lettering is shown when *p* < 0.05.

ALT, alanine transaminase; AST: aspartate transaminase; BMI: body mass index; HDL: high‐density lipoprotein; HOMA‐IR: homeostasis model assessment of insulin resistance; LDL: low‐density lipoprotein; MCP‐1, monocyte chemoattractant protein‐1; TNFα: tumor necrosis factor‐α.

We obtained subcutaneous adipose tissue biopsies from elderly (*n* = 13) and young control (*n* = 10) individuals and analyzed several components of the FGF21‐responsive molecular machinery. We found that expression of the β‐Klotho gene was significantly increased in elderly individuals compared with young healthy controls (Figure 1b). Expression of the gene for FGF receptor‐1 (FGFR1), the main FGF receptor that mediates FGF21 action in adipose tissue (Kurosu et al., 2007), was not changed. In line with gene expression results, β‐Klotho protein levels, measured by immunoblotting, were higher in subcutaneous adipose tissue from elderly individuals compared with those from healthy young controls (Figure 1c). Moreover, decreased ERK1/2 protein levels in elderly individuals were accompanied by an increase in the extent of ERK1/2 phosphorylation, resulting in a significantly higher ratio of phospho‐ERK1/2 to total ERK1/2 protein in adipose tissue from elderly individuals than in those from young controls (Figure 1c). These results suggest that abnormally elevated FGF21 levels in healthy aging are not associated with repressed FGF21‐responsiveness machinery of FGF21 responsiveness in adipose tissue, a finding that contrasts with what is observed in obesity and diabetes (Gallego‐Escuredo et al., 2015).

Considering that ethical and practical issues precluded the functional analysis of FGF21 effects on fresh adipose tissue from aged healthy humans, a mouse model was used. Aged mice (16‐month‐old) showed a profile of alterations relative to young (5‐month‐old) mice that is in general consistent with human data: Plasma FGF21 levels were increased, whereas there were no signs of impaired expression of mediators of FGF21 signaling such as FGFR1 and β‐Klotho (see Supporting Information Appendix S1). Treatment of adipose tissue explants with FGF21 in vitro caused a significant induction in the ratio of phospho‐ERK1/2 to total ERK1/2, as well as of expression of Egr1 and c‐Fos, early‐responsive genes to FGF21 (Fisher et al., 2010). The extent of FGF21 effects was not different in adipose tissue from aged and young mice (Figure 1d) thus supporting unaltered responsiveness of aged adipose tissue to FGF21, as suggested by human data.

An increase in systemic and local inflammation with aging has been reported (Cevenini et al., 2010). High tumor necrosis factor (TNF)‐α levels and local inflammation have been shown to inhibit β‐Klotho expression in adipose tissue (Diaz‐Delfin et al., 2012), and it has been hypothesized that a local proinflammatory environment is responsible for β‐Klotho downregulation in obesity. Consistent with this, we found that plasma levels of the proinflammatory cytokines, TNFα and monocyte chemoattractant protein‐1 (MCP‐1), were significantly higher in healthy elderly individuals than in young controls (Table 1). Moreover, expression of TNFα and MCP‐1 genes in adipose tissue was increased in healthy elderly individuals compared with young controls (Figure 1b). Thus, locally increased expression of TNFα in aging does not appear to lead to impaired β‐Klotho expression in adipose tissue. In this context, a correlation analysis revealed a positive correlation of systemic levels of TNFα (*r* = 0.376 and *p* = 0.017) with circulating levels of FGF21 in the studied cohort.

Collectively, our results suggest that healthy aging is not accompanied by impaired responsiveness of subcutaneous adipose tissue to FGF21. Even if healthy elderly individuals suffered local (adipose tissue) and systemic inflammation, β‐Klotho expression was not impaired by proinflammatory signals. Moreover, and contrary to expectations, expression of β‐Klotho and markers of FGF21 action in this FGF21‐target tissue actually increased. Our findings also confirm that healthy human aging is associated with increased FGF21, despite the appearance of mild signs of distorted glucose homeostasis and increased inflammation associated with healthy aging; in fact, it is likely that these metabolic stressors are involved in the increase in FGF21. Thus, either FGF21 resistance per se does not occur during aging or tissues other than subcutaneous fat are the actual source of such resistance. Identification of the brain as a target of FGF21 (Kuroda et al., 2017) highlights the potential resistance to FGF21 in the brain as a particularly relevant concept in further aging research. In either case, these results contrast with the situation that prevails in obesity, diabetes, and lipodystrophy (Gallego‐Escuredo et al., 2012, 2015 ). As a result, further intervention‐based studies would be required to fully determine systemic and tissue‐specific sensitivity to FGF21 in human aging.

There are some obvious limitations to our study. The numbers of individuals in our cohorts were not high. For example, a separate analysis of young and elderly individuals by biological sex did not reveal sex‐dependent effects on any of the parameters tested (Supporting Information Appendix S1), but studies on a larger number of individuals could reinforce this negative observation. Moreover, visceral adipose tissue, a type of fat targeted by FGF21 that is especially sensitive to metabolic disease‐associated alterations, and liver, could not be studied for the obvious reasons that such biopsies cannot ethically be obtained from aged healthy volunteers. Further research will be needed to determine whether other tissues follow the same pattern of FGF21 responsiveness as subcutaneous adipose tissue. At last, functional assessment of unaltered effects of FGF21 in adipose tissue in aged individuals relied on a mouse model. In any case, ours is the first report that age‐related increases in FGF21 are not associated with an impairment in the FGF21‐responsive machinery in adipose tissue and establish a distinction between alterations in the FGF21 endocrine system in aging and those in obesity and diabetes.

## CONFLICT OF INTEREST

All authors have nothing to disclose.

## **AUTHOR** **CONTRIBUTION**

J.V. and J.M.G‐E performed transcript and proteins measurements in adipose tissue biopsies and FGF21 and FGF19 quantification in serum; R.M. and J.C.D. performed analytical procedures in serum; M.G.M. and P.D. recruited and characterized the population of individuals to be analyzed; F.V., M.C., and A.D. performed functional assays in adipose tissue explants; M.G.M. performed blood extraction and adipose tissue biopsies; P.D., M.G., and F.V. analyzed the data; M.G. and F.V. wrote the manuscript. All authors reviewed the manuscript and contributed to overall discussion of the data.
