# Supplementary material for: Aging is associated with increased FGF21 levels but unaltered FGF21 responsiveness in adipose tissue
Source: Aging Cell. 2018 Jul 24;17(5):e12822. doi: 10.1111/acel.12822 (PMC6156525; doi:10.1111/acel.12822)
Supplement: Supplementary file 1 [file ACEL-17-e12822-s001.docx]

**Supporting Information.**

**Experimental Procedures**

*Cohort study and demographic data*

This study was approved by the ethics committee of Hospital de la Santa Creu i Sant Pau, Barcelona. A total of 28 healthy individuals over 70 year-old and 35 healthy controls were enrolled in this cross-sectional study. To consider an old patient healthy, no evidence of any chronic disease had to be present, including inflammatory diseases, cardiovascular disease, hepatitis, liver insufficiency, fever of undetermined origin, diabetes mellitus, and cancer. Only arterial hypertension and antihypertensive therapy were allowed. Exclusion criteria also included, body mass index (BMI) > 30 kg/m^2^, anticoagulant treatment, oral antidiabetic therapy, and hormonal treatment.

All patients provided informed written consent. Demographics, body composition and metabolic markers are shown in Table 1. BMI was calculated, and waist circumference was measured to the nearest millimeter using anatomical landmarks, as defined by the Third National Health and Nutrition Evaluation Survey. Whole-body, dual-energy X-ray absorptiometry (DEXA) scans (HologicQDR-4500A; Hologic, Inc., Waltham, MA, USA) were conducted by a single operator and used to determine body fat content. Plasma and serum were obtained from blood drawn from seated patients after a 12-hour overnight fast and at least 15 minutes after the placement of a peripheral intravenous catheter. All lipid measurements were performed using a Hitachi 911 system (Roche Diagnostic Systems, Basel, Switzerland). Insulin resistance was estimated by the homeostasis model assessment method (HOMA-IR). Laboratory procedures have been described elsewhere (Domingo et al., 2015).

*Systemic parameter detection*

Serum FGF21 and FGF19 levels were determined in duplicate for each sample using enzyme-linked immunosorbent assays (ELISA) specific for human FGF21 and FGF19 (Biovendor, Czech Republic). Serum FGF21 and FGF19 data distribution was skewed and was thus log-transformed before analysis.

Circulating TNFα and MCP-1 levels in serum were measured using an antibody-linked, fluorescently labeled microsphere bead-based multiplex analysis system (Linco Research/Millipore, Billerica, MA, USA) and quantified using Luminex100ISv2 equipment.

*Biopsy samples and protein measurement*

Biopsy samples of subcutaneous fat from abdominal area from lean healthy controls (n=10) and healthy elderly individuals (n=13) were collected from patients through a small surgical biopsy performed by an 8 mm punch under local anesthesia with mepivacaine. Tissue samples were immediately frozen and stored at -80°C. After homogenization in RLT buffer (Qiagen, Hilden, Germany), RNA was isolated using a column-affinity based methodology that included on-column DNA digestion (RNeasy; Qiagen). One microgram of RNA was transcribed into cDNA using MultiScribe reverse transcriptase and random-hexamer primers (TaqMan Reverse Transcription Reagents; Applied Biosystems, Foster City, California, CA, USA). For quantitative mRNA expression analysis, TaqMan reverse transcriptase (RT)-polymerase chain reaction (PCR) was performed on the ABI PRISM 7700HT sequence detection system (Applied Biosystems). The TaqMan RT-PCR reactions were performed in a final volume of 25 µl using TaqMan Universal PCR Master Mix, No AmpErase UNG reagent and primer pair probes specific for FGFR1 (Hs00222484_m1), β-Klotho (Hs00545621_m1), TNFα (Hs00174128_m1), MCP-1 (Hs00234140_m1) and 18S rRNA, (Hs99999901). Controls with no RNA, primers, or RT were included in each set of experiments. Each sample was run in duplicate, and the mean value of the duplicate was used to calculate the mRNA levels for the genes of interest. Expression levels of gene transcripts were considered negligible when, under the above standard RT-PCR conditions, cycle threshold was > 40. Values were normalized to that of the reference control (18S ribosomal RNA) using the comparative 2^-ΔCT^ method, following the manufacturer's instructions. Parallel calculations performed using the reference gene PPIA (Hs99999904) yielded essentially the same results.

For quantification of protein levels, adipose tissue samples were homogenized in cold buffer (10 mM HEPES pH 7.5, 5 mM EDTA, 5 mM dithiothreitol, 5 mM MgCl_2_), and a cocktail of protease inhibitors (Complete-mini, Roche, Sant Cugat, Spain). For Western blot analysis, homogenates containing 40 μg of protein were mixed with equal volumes of 2 x sodium dodecyl sulfate (SDS) loading buffer, incubated at 90ºC for 5 min, and electrophoresed on SDS/polyacrylamide gels. After transferring to Immobilon-P membranes (Millipore, Billerica, MA, USA), proteins were probed using antibody directed against β-Klotho (ab76356) (Abcam, Cambridge, UK), Phospho-ERK1/2 and total ERK1/2 (Cell Signaling Technology, Danvers, MA, USA). Goat anti-rabbit HRP-conjugated antibody (Santa Cruz Biotechnology, Dallas, Texas, USA) and ECL reagents (Immobilon Western; Millipore, Germany) were used to detect the immunoreactive signals. Membranes were stained with Coomassie blue (Sigma-Aldrich, St Louis, MO, USA) to normalize the amount of protein loaded. Multi-Gauge software (Fujifilm) was used for densitometric analyses.

Six individual samples for each group were analyzed. Representative pictures (additional to those shown in Fig 1) are shown:


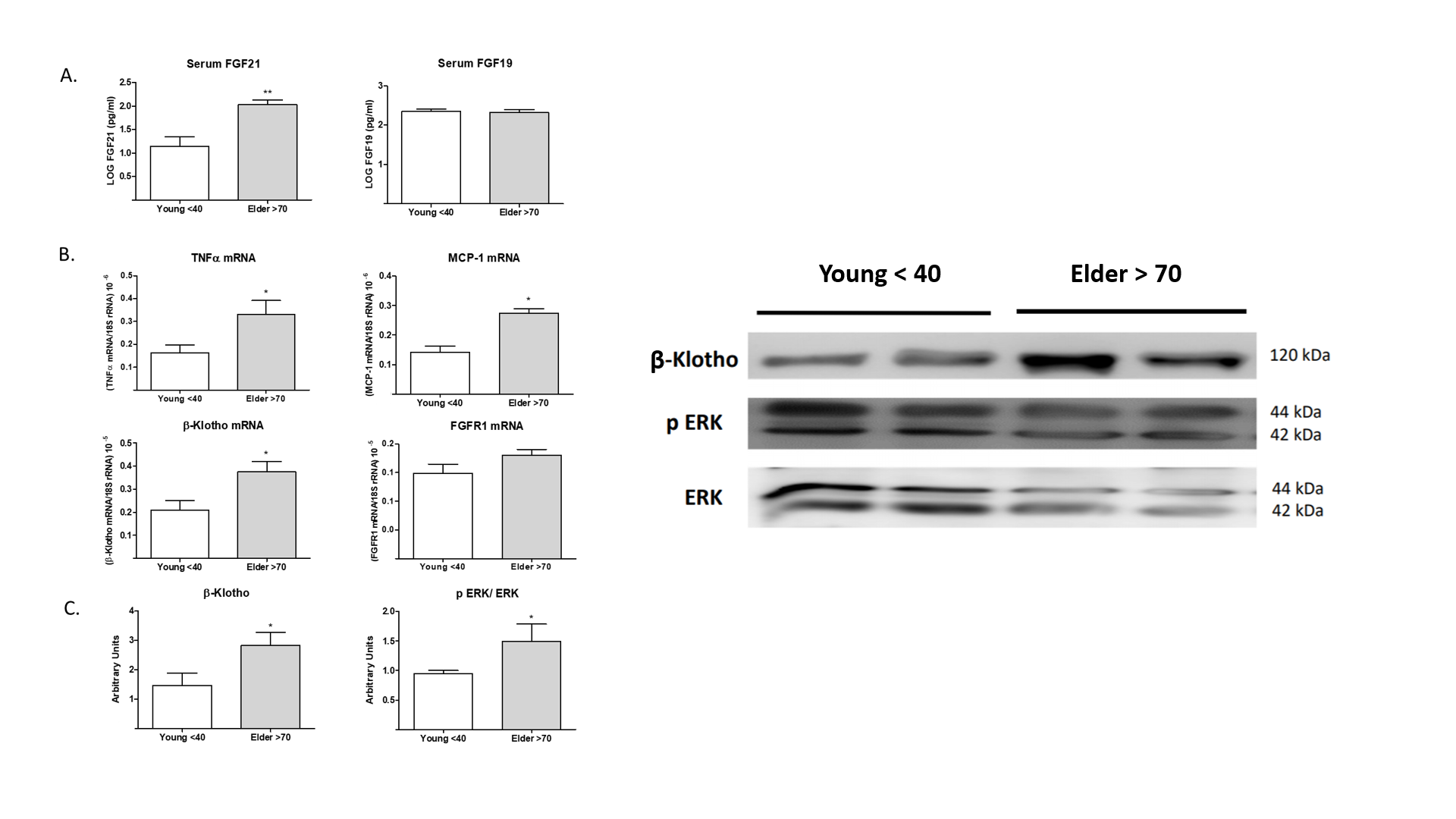


*Mouse adipose tissue study*

Adult young (5 month-old) and aged (16 month-old) C57/BL6 mice (Harlan Laboratories, Sant Feliu de Codines, Spain) were sacrificed. Blood was obtained and adipose tissue explants were obtained and incubated in Dulbecco’s modified Eagle Medium, 1.5 g/l glucose for 15 or 30 min in the presence (or absence) of 30 nM FGF21 (Phoenix Pharmaceuticals, Germany). The remaining tissue was frozen for further analysis. The following gene transcripts were measured in frozen tissues or explants, using the qRT-PCR methodology as for human samples (see above), but using the mouse-specific TaqMan probes: FGFR1, (Mm00438930_m1), β-Klotho (Mm00473122_m1), Egr1 (Mm00656724_m1), c-Fos (Mm00487425_m1). P-ERK and ERK levels were determined using immunoblot with mouse/human cross-reactive antibody (Cell Signaling Technology, Danvers, MA, USA). These studies were approved by the Institutional Animal Care and Use Committee of the University of Barcelona.

Four individual mice were used and assays were performed in triplicate. Representative pictures are shown:


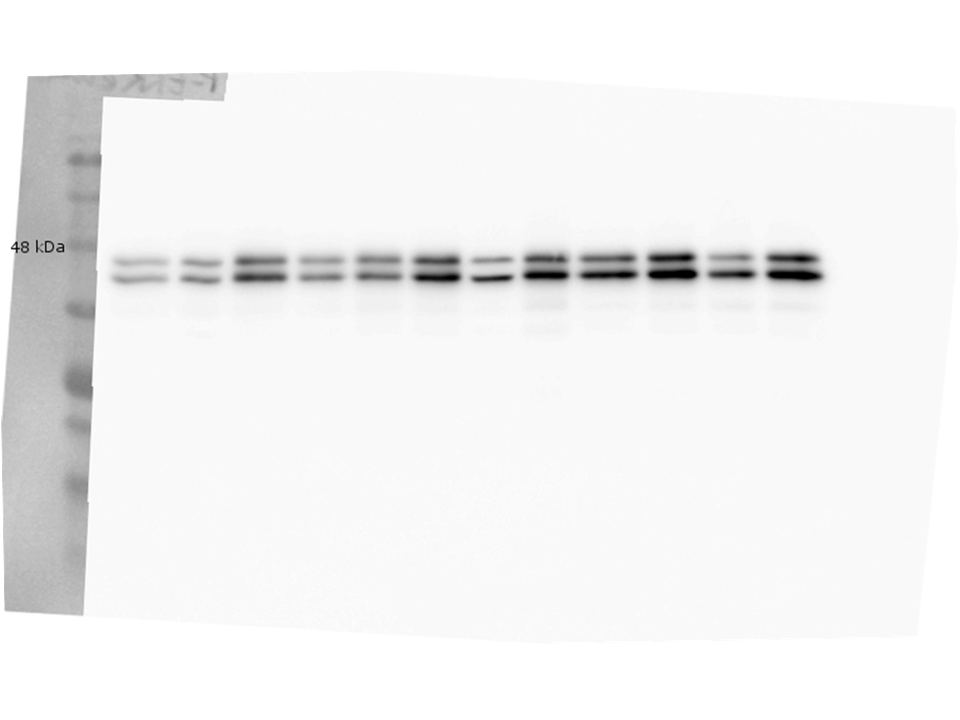


**FGF21:**

- + - + - + - +

**Young**

**Aged**


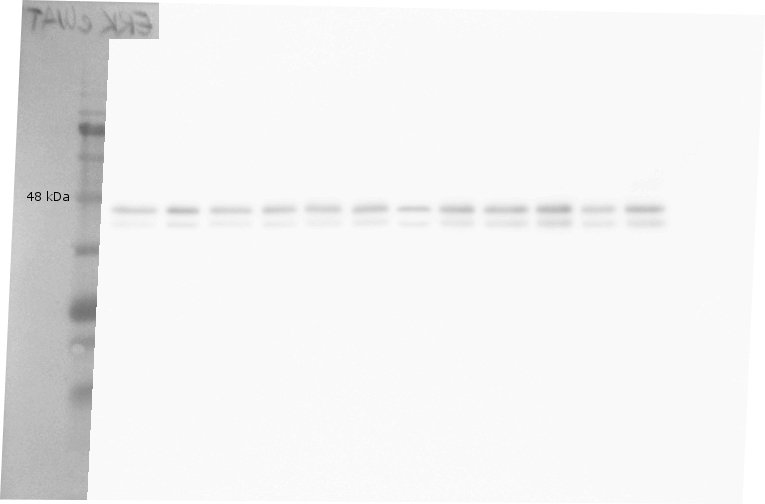


**p ERK**

**ERK**

**44 kDa**

**42 kDa**

**44 kDa**

**42 kDa**

*Statistical analysis*

Data were expressed as means ± standard errors of the mean (SEMs), frequencies, or percentages relative to healthy controls (defined as 100%). The normality of parameter distributions was determined using a Kolmogorov-Smirnov analysis and data were normalized by log-transformation when needed. Student T-test were used for comparisons of parametric data. Correlation analysis was used to determine the linear relationships of anthropometric, metabolic, and other variables with quantitative serum FGF21 parameters. Statistical analyses were performed using the Statistical Package for Social Sciences version 17.0 (SPSS, Chicago, IL, USA) and SAS version 9.1.3 software (SAS Institute Inc., Cary, NC, USA). P-values < 0.05 (determined by two-sided tests) were considered significant.

References:

Domingo P, Lamarca MK, Gallego-Escuredo JM, Torres F, Domingo JC, Villarroya J, Gutierrez Mdel M, Mateo MG, Vidal F, Villarroya F, Giralt M (2015). Circulating fibroblast growth factor 23 (FGF23) levels are associated with metabolic disturbances and fat distribution but not cardiovascular risk in HIV-infected patients. J Antimicrob Chemother. 70, 1825-1832.

**Supplementary Table 1.** Serum and mRNA levels in adipose tissue from young healthy controls and elderly individuals separated by sex

| **Serum levels** | **Young**  **Male (23)** | **Young female (12)** | P value male vs female | **Elderly male (14)** | **Elderly female (14)** | P value male vs. female |
| --- | --- | --- | --- | --- | --- | --- |
| FGF21 (pg/ml) | 57 + 11 | 46 + 10 | 0.47 | 132 + 23 | 180 + 34 | 0.28 |
| FGF19  (pg/ml) | 266+ 31 | 250 + 64 | 0.83 | 495 + 99 | 375 + 114 | 0.44 |

| **mRNA/**  **18S rRNA** | **Young**  **Male (5)** | **Young female (5)** | P value male vs female | **Elderly male (7)** | **Elderly female (6)** | P value male vs. female |
| --- | --- | --- | --- | --- | --- | --- |
| β-Klotho | 1.81 + 0.64  x 10^-5^ | 2.59 + 0.79  x 10^-5^ | 0.46 | 3.56 + 0.71  x 10^-5^ | 4.04 + 0.88  x 10^-5^ | 0.57 |
| MCP-1 | 1.77 + 0.68  x 10^-5^ | 1.41 + 0.22  x 10^-5^ | 0.77 | 2.39 + 0.37  x 10^-5^ | 3.01 + 0.41  x 10^-5^ | 0.26 |
| TNFα | 1.81 + 0.85  x 10^-6^ | 1.91 + 0.51  x 10^-6^ | 0.95 | 2.88 + 0.70  x 10^-6^ | 3.61 + 0.81  x 10^-6^ | 0.54 |
| FGFR1 | 1.39 + 0.57  x 10^-4^ | 1.71 + 0.21  x 10^-4^ | 0.68 | 1.64 + 0.23  x 10^-4^ | 1.87 + 0.48  x 10^-4^ | 0.69 |

**Supplementary Table 2.** Serum and adipose tissue mRNA levels in young and aged mice.

| **Serum levels** | **Young mice**  **(5 month-old)** | **Aged mice**  **(16 month-old)** | P value |
| --- | --- | --- | --- |
| FGF21 (pg/ml) | 107 ± 41 | 524 ± 49 | 0.0003 |

| **mRNA/**  **18S rRNA** | **Young mice**  **(5 month-old** | **Aged mice**  **(16 month-old** | P value |
| --- | --- | --- | --- |
| β-Klotho | 4.76 ± 0.63 x10^-5^ | 3.85 ± 0.45 x10^-5^ | 0.29 |
| FGFR1 | 9.25 ± 1.75 x10^-5^ | 9.43 ± 0.87 x10^-5^ | 0.93 |
